# Oral health status and hygiene practices among visually impaired adolescents from a school in Kenya

**DOI:** 10.1186/s12903-023-03428-7

**Published:** 2023-10-07

**Authors:** Maureen Macharia, Mary Masiga, Nathan Psiwa, Janella Bermudez, Ana Lucia Seminario, Arthur Musakulu Kemoli

**Affiliations:** 1 Department of Pediatric Dentistry and Orthodontics, School of Dental Sciences, University of Nairobi, Nairobi, Kenya; 2 Advanced Education in General Dentistry Program, Yakima, Washington, USA; 3 School of Dentistry, School of Public Health, University of Washington, Seattle, USA

**Keywords:** Oral health status, Oral hygiene practices, Visual impairment, Adolescents, Kenya

## Abstract

**Background:**

Visual impairment affects a significant population globally. The aim of this study was to determine the oral health status and oral hygiene practices among visually impaired adolescents from a school in Kenya.

**Methods:**

A descriptive cross-sectional study was carried out among 159 adolescents aged 10–19 years attending the largest public primary boarding school for the blind in Kenya. A questionnaire was used to record participants’ socio-demographic variables and oral hygiene practices. Clinical examination was undertaken to assess oral health status which consisted of oral hygiene, gingival health, and dental caries. Bivariate analyses were conducted to compare dental health outcomes across socio-demographic characteristics.

**Results:**

There were 69 (43.4%) and 90 (56.6%) participants in Category I and II visual impairment respectively, 85 (53.5%) were male and 74 (46.5%) were female. Study participants were divided into three age categories: 10–12 years 48 (30.2%), 13–15 years 67 (42.1%), and 16- 19 years 44 (27.7%), with an overall mean age of 13.9 ± 2.3. All participants brushed their teeth, majority 107 (67.3%) brushed two or more times daily. Only 66 (41.5%) of the participants replaced their toothbrushes at 3 months. Sex (*p* =< 0.001) and age (*p* = 0.04) influenced frequency of toothbrush replacement. The average plaque score and gingival score index was 0.95 ± 0.45 and 0.28 ± 0.25 respectively, with gingivitis prevalence of 88.1%. Overall dental caries prevalence was 44.7%, [42.1%)] permanent dentition and [8.2%] deciduous dentition. Mean DMFT and dmft were 0.44 ± 0.60 and 0.12 ± 0.32 respectively. DMFT had a statistically significant association with sex (t = 1.82, *p* = 0.03). Oral hygiene practices did not influence oral hygiene and dental caries status. However, a statistically significant association was reported between frequency of toothbrush replacement and gingival score index (“*p*” =< 0.001).

**Conclusions:**

The study reported general good oral hygiene, prevalent gingivitis 140 (88.1%), and almost half of the study population affected by dental caries 71 (44.7%). Most participants were unaware of using fluoridated toothpaste and of needing to change toothbrushes within 3 months. Frequency of toothbrush replacement was reported to influence gingival score index.

## Background

Visual impairment is a sensory deterioration recognized as a major public health concern and is ranked sixth in the global burden of disease in relation to disability-adjusted life-years [1, 2]. It ranges from low vision to total blindness, and the World Health Organization (WHO) has classified it in terms of visual acuity as mild (< 6/12–6/18), moderate (< 6/18–6/60), severe (< 6/60–3/30), and blind (< 3/60) [3]. This means that a blind person with a visual acuity of < 3/60 would have to stand 3 m away to see what a person with normal vision would see at 60 m. Globally, a total of 2.2 billion people are affected by visual impairment [3], of these, 14 million children are blind [4]. The majority (75%) of these children live in the poorest regions in Africa and Asia [5]. In Kenya, the prevalence of pediatric visual impairment has been reported to be 3.1% [6]. Accurate measurements of visual acuity are important as they serve to determine eligibility for government support in some countries [7]. A good example is an access to education in the United States of America for children with special health needs that focuses on self-care, social skills, and vocational training [7]. However, in school, it is more useful for educators to classify visual impairment based on students’ ability to use their visual and other channels, such as tactile and auditory senses for learning [8]. The Low Vision Project – Kenya classifies students with visual impairment into five groups [9]. Students in category I are totally blind, have no perception of light, and are educated in Braille. Students in category II have low vision, which is not adequate to read print, hence, are educated in Braille. Students in category III have low vision and can be trained to use optical low-vision devices such as magnifying glasses to read and write print. Students in category IV have low vision and can be educated in print using special methods such as the use of large print but without the use of optical low-vision devices. Students in category V are not low visioned as their sight is above 6/18 and do not need special education if their sight remains constant [9].

Good oral health constitutes a key aspect of general health and the quality of life of the individual [10]. Oral hygiene practices are associated with oral diseases, such as dental caries, and play a major role in the overall dental health of an individual. Nonetheless, maintenance of good oral health is particularly challenging for the visually impaired and has been reported to be poor in comparison to the general population [5, 11]. Related factors include difficulty in attaining good oral hygiene and the inapplicability of visual aids used in the demonstration of oral hygiene instructions [12, 13]. Challenges for maintaining adequate oral health among visually impaired children can be aggravated in low- and middle-income countries due to limited access to oral healthcare. In Kenya, there is a high burden of oral diseases among the pediatric population. Dental caries and gingival bleeding prevalence among children and adolescents (5, 12, and 15 year - olds) have been reported to be 23.9% and 75.7%, respectively [14]. However, there is scarce evidence, especially in Sub-Saharan countries, including Kenya, on oral health status and oral hygiene practices among visually impaired children and adolescents [15].

The purpose of the study was to answer the following question: ‘Among adolescents with category I and II visual impairment who attended the largest public primary boarding school for the Blind in Kenya, did oral hygiene practices influence oral hygiene status’. We hypothesized that good oral hygiene practices were associated with a lower prevalence of oral diseases. The specific aim of the study was to determine the prevalence of dental caries and gingivitis, evaluate oral hygiene status, and relate these factors to the oral hygiene practices among visually impaired adolescents attending Thika Primary School for the Blind in Kiambu County, Kenya. Results from this study will provide baseline data and inform relevant health planners in the formulation of oral health programs for visually impaired children with the aim of promoting and providing continuous and sustainable oral health care.

## Methods

**Fig. 1:**
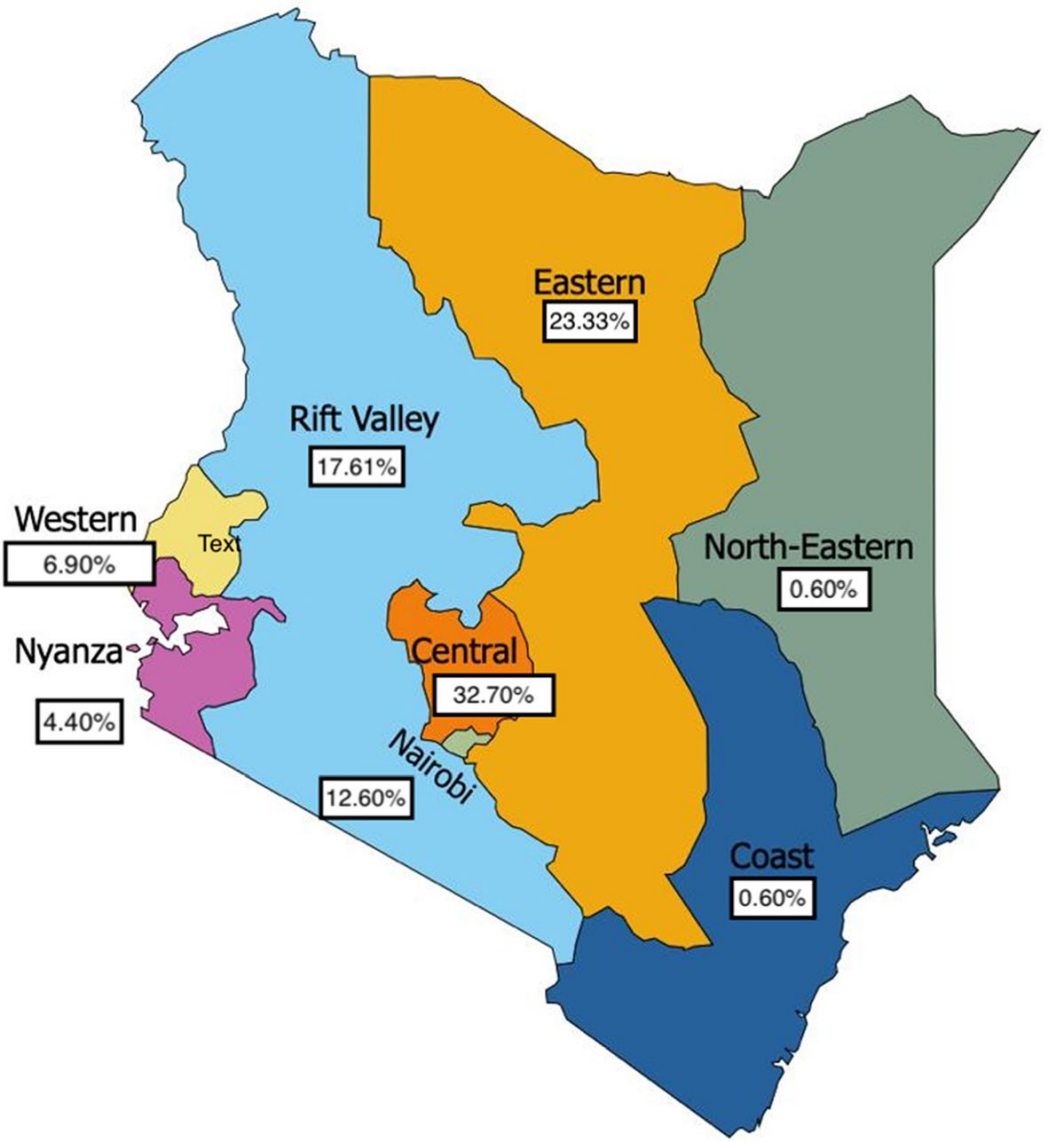
Map of Kenya showing the distribution of participants by regions

Prior to commencement of this study, ethical clearance was obtained from the relevant body.This was a descriptive cross-sectional study conducted at the Thika Primary School for the Blind in Kiambu County, Central Kenya. The school is the largest of the four institutionalized public primary schools serving children with visual impairment in the country. Kenya is comprised of 47 counties, however, for purposes of this study, the counties were stratified into 8 geographical regions (Fig. 1): region 1 (Coast), region 2 (North Eastern), region 3 (Eastern), region 4 (Central), region 5 (Rift Valley), region 6 (Western), region 7 (Nyanza), and region 8 (Nairobi) [16].

The study participants were aged 10–19 years old based on the definition of adolescents by the United Nations Children’s Fund (UNICEF). The sample size was determined using the formula proposed by Fisher et al. [17]. Assuming visually impaired children with dental caries to be 46.8% [11] and considering a 95% confidence level and 5% degree of accuracy. An estimated population size of 209 was used and the minimum sample size was computed to 148 after adjusting for 10% attrition. However, all participants who met the inclusion criteria were included in the study and the final sample size was 159, representing 60% of the school population.

Proportionate, stratified random sampling was employed in the recruitment of study participants. The study population was stratified into three age groups: mixed dentition consisting of 10–12 years old and permanent dentition consisting of 13–15 and 16–19 years old. In each stratum, an alphabetical listing of names was obtained and numbered serially, and random numbers were generated by the computer that was used to select the requisite number of individuals in each stratum. Subjects eligible for the study inclusion were adolescents aged 10–19 years with visual educational categories I and II, who used Braille as their mode of learning, assented to the study, and whose parents consented to the study. Subjects were excluded from the study enrolment if they had physically or mentally debilitating conditions (E.g., Cerebral Palsy or syndromes such as Down’s syndrome) which may have impacted oral health status and skill to carry out oral hygiene practices, or if they did not consent to the study.

### Data collection

Calibration for the principal investigator (PI) was carried by an experienced pediatric dentist at Lady Northey Dental Hospital. A modified questionnaire adopted from the Simplified Oral Health Questionnaire for Children World Health Organization was used [18]. The questionnaire contained both open and closed-ended questions and was used to record participants’ socio-demographic variables and oral hygiene practices. It was pre-tested on adolescents aged between 10–19 years at the University of Nairobi Dental Hospital, to check the suitability, simplicity, and ease of understanding, as well as to estimate the time taken to complete the questionnaire. It was then administered by the PI to the study participants before a clinical examination of the participants was done.

A clinical examination was undertaken by the PI to assess oral health status which consisted of oral hygiene, gingival health, and dental caries. The clinical findings were recorded in a modified WHO Oral Health Assessment Form for Children by a trained data clerk assistant [18]. Oral hygiene status was assessed first using the plaque index score described by Silness and Löe [19]. Plaque score findings were classified as 0- no plaque; 1- film of plaque adhering to the free gingival margin and adjacent area of the tooth; 2- moderate accumulation of the soft deposits within the gingival pocket or the tooth and gingival margin; 3-abundance of soft matter within the gingival pocket and or on the tooth and gingival margin. Gingival health was assessed after the plaque score, using the Community Periodontal Index (CPI) Modified [18]. Briefly, the WHO CPI dental probe was gently inserted between the gingiva and the tooth to explore the full extent of the sulcus. Gingival score findings were categorized as presence or absence of bleeding. Dental caries was determined using visual and tactile examination. Individual teeth were isolated and dried using sterile gauze in a systematic pattern from one tooth to the adjacent one in each quadrant. Each tooth was recorded as decayed/Decayed, missing/Missing, or filled/Filled due to caries; [dmft (for primary dentition) /DMFT (for permanent dentition)] [18].

### Data analysis

The data which had been recorded on paper forms was later entered into a computer database and analyzed using Statistical Package for Social Sciences (SPSS) version 23.0 of Windows. Characteristics of the study population were summarized using descriptive statistics. Analysis of Variance (ANOVA) was used to test differences between oral health status (plaque and gingival scores and dental caries) by age group with Bonferroni correction test used to mitigate the risk of false discovery. An independent samples t-test was used to test for a statistically significant difference between oral health status by the category of visual impairment and by sex. Pearson’s Chi-square was used to assess bivariate relationships between oral hygiene practices by sex, age group and category of visual impairment and between category of visual impairment by sex and age group. Fisher’s exact test was used to determine associations where cells had frequencies less than 5. Spearman’s correlation was used to assess associations between oral hygiene practices and oral health status. The critical value was set at 5%.

## Results

### General characteristics

An estimated population size of 209 was used during the study and the sample size was computed to 148. During calibration of the PI, mean Cohen Kappa statistic values obtained for inter-examiner reliability were, plaque score = 0.85, gingival index = 0.88, dmft = 0.90 and DMFT = 0.89; values obtained for intra-examiner reliability were, plaque score = 0.95, gingival index = 1.00, dmft = 0.87 and DMFT = 0.82.

There were 69 (43.4%) and 90 (56.6%) participants in Category I and II visual impairment respectively. Of the total participants, 85 (53.5%) were male and 74 (46.5%) were female. The participants were divided into three age categories: 10–12 years (30.2%, *n* = 48,), 13–15 years (42.1%, *n* = 67), and 16–19 years (27.7%, *n* = 44,), with an overall mean age of 13.9 ± 2.3 (Table 1). The Central region of the country had the largest representation (54 or 32.7%), while the Coastal and North-Eastern regions had the least representation (1 or 0.6%) (Fig. 1).

**Table 1:** Demographic characteristics of the study population

**Demographic**	***N* = 159 (%)**
**Visual impairment**	
Category I	69 (43.4%)
Category II	90 (56.6%)
**Sex**	
Male	85 (53.5%)
Female	74 (46.5%)
**Age**	
10–12 yrs	48 (30.2%)
13–15 yrs	67 (42.1%)
16–19 yrs	44 (27.7%)
**Mean Age (SD)**	**13.9 ± 2.3**

### Oral hygiene practice

All participants reported to brush their teeth, majority [67.3%, (*n* = 107)] brushed two or more times daily, while [32.7%, (*n* = 52)] brushed less than twice a day. All participants utilized commercial toothbrushes, with [41.5%, (*n* = 66)] replacing the toothbrushes at 3 months. There was a statistically significant association between frequency of toothbrush replacement, age (*p* =< 0.001) and sex (*p* = 0.04). Other adjunct devices used or toothbrushing included wooden toothpicks [62.9%, (*n* = 100)] and chewing sticks/ “mswaki” [25.2%, (*n* = 40)]. Most [99.4%, (*n* = 158)] of the participants reported using toothpaste with the majority [93.1%, (*n* = 148)] unaware if the toothpaste they used contained fluoride. Most of the participants [(86.7%, (*n* = 137)] rinsed their mouth with water after meals, while [6.3%, (*n* = 10)] seldom rinsed, and [(7%, (*n* = 11)] did not rinse at all (Table 2). A statistically significant correlation was reported between frequency of toothbrush replacement and gingival index score (“*p*” =< 0.001) (Table 3).

**Table 2:** Oral hygiene practices in relation to sex and age

**Oral Hygiene practices**	**Sex**		***P*-value**	**Age (yrs)**			***P*-value**
	**Male**	**Female**		**10–12**	**13–15**	**16–19**	
	**N (%)**			**N (%)**			
**Frequency of tooth brushing**							
Two or more times a day	55 (64.7%)	52 (70.3%)	0.72	29 (60.4%)	47 (70.1%)	31 (70.5%)	0.59
Less than twice daily	30 (35.5%)	22 (29.8%)		19 (39.6%)	20 (29.9%)	13 (29.5%)	
**Adjunct toothbrush devices**							
Wooden toothpicks	40 (40%)	60 (60%)	0.42	20 (26.0%)	27 (27%)	47 (47%)	0.74
Plastic Toothpicks	0 (0%)	2 (100%)	0.34^a^	0 (0%)	1 (50%)	1 (50%)	0.48^a^
Dental Floss	5 (62.5%)	3 (37.5%)	0.57	1 (2.5%)	2 (25%)	5 (62.5%)	0.08
Charcoal	4 (44.4%)	5 (55.6%0	0.10	2 (22.2%)	4 (44.4%)	3 (33.3%)	0.31
Chew stick/mswaki	19 (47.5%)	21 (52.5%)	0.50	10 (25%)	18 (45%)	12 (30%)	0.74
**Use of fluoridate toothpaste**							
Yes	2 (2.4%)	2 (2.7%)	0.97^a^	1 (2.1%)	1 (1.5%)	2 (4.5%)	0.50^a^
No	4 (4.7%)	3 (4.1%)		1 (2.1%)	5 (7.5%)	1 (2.3%)	
Don’t know	79 (92.9%)	69 (93.2%)		46 (95.8%)	61 (91%)	41 (93.2%)	
**Mouth rinsing after meals**							
Yes	72 (84.7%)	65 (89%)	0.71^a^	45 (93.8%)	57 (85.1%)	35 (81.4%)	0.12^a^
No	7 (8.2%)	4 (5.5%)		1 (2.1%)	6 (9.0%)	4 (9.3%)	
Seldom	6 (7.1%)	4 (5.5%)		2 (3.04%)	4 (42.4%)	4 (27.2%)	
**Frequency of toothbrush replacement**							
< 3 months	4 (4.7%)	9 (12.2%)	0.04*^a^	0 (0%)	7 (10.4%)	6 (13.9%)	< 0.001*
3 months	37 (43.5%)	29 (39.2%)		14 (29.2%)	31 (46.3%)	21 (47.7%)	
> 3 months	39 (45.9%)	24 (32.4%)		24 (32.4%)	21 (47.7%)	16 (36.4%)	
Not sure	5 (5.9%)	10 (20.8%)		10 (20.8%)	16 (36.4%)	19 (2.3%)	

^*^p ≤ 0.05

^a^Fisher exact test

**Table 3:** Correlation matrix for oral hygiene practices and oral health status

**Oral hygiene practices**	**Gingival Score**		**Plaque Score**		**DMFT**		**dmft**	
	***p*-value**	**rs-value**	***p*-value**	**rs-value**	***p*-value**	**rs-value**	***p*-value**	**rs-value**
**Frequency of toothbrushing**	0.31	0.12	0.42	0.02	0.53	1.00	0.09	0.01
**Adjunct toothbrush devices**								
Wooden toothpicks	0.15	0.08	0.81	0.02	0.09	0.04	0.06	0.01
Plastic Toothpicks	0.93	0.04	0.45	0.06	0.58	0.01	0.20	0.04
Dental Floss	0.06	0.13	0.34	0.07	0.05	0.04	0.18	0.05
Charcoal	0.13	0.20	0.53	0.05	0.06	0.05	0.20	0.04
Chew stick/mswaki	0.09	0.01	0.70	0.03	0.23	0.09	0.13	0.09
**Use of fluoridate toothpaste**	0.76	0.18	0.08	0.09	0.27	0.06	0.25	0.06
**Mouth rinsing after meals**	0.13	0.06	0.08	0.08	0.20	0.09	0.18	0.09
**Frequency of toothbrush replacement**	< 0.001*	0.50	0.05	0.05	0.51	0.08	0.08	0.08

^*^p ≤ 0.05

### Oral hygiene status and gingival health

The average plaque score among all participants was 0.95 ± 0.45. Female participants plaque score value was 0.88 ± 0.44 while that of the male participants was (1.02 ± 0.45). Among participants in category 13–15 years old, plaque score was (0.99 ± 0.47) and (0.87 ± 0.44) among those in category 10–12 years old. The overall prevalence of gingivitis was 88.1% with a mean gingival score index of 0.28 ± 0.25. Gingival scores significantly varied by age (*p* = 0.02). Adolescents aged 16–19 years had the highest gingival score index (0.34 + 0.28), while those aged 10–12 years had the least gingival score index (0.20 + 0.21) (Table 4). Plaque score index and gingival score index were moderately correlated; with a coefficient of (rs = 0.52) (*p* =< 0.001).

**Table 4:** Distribution of participants by oral hygiene status and gingival inflammation

**Characteristics**	***N* = 159 (%)**	**Plaque score**		**Gingival score**	
		**(M ± SD)**	***p*-value**	**(M ± SD)**	***p*-value**
**Sex**					
Male	85 (53.5%)	1.02 ± 0.45	0.88	0.30 ± 0.25	0.17
Female	74 (46.5%)	0.88 ± 0.44		0.25 ± 0.24	
**Age**					
10–12 yrs	48 (30.2%)	0.87 ± 0.44	0.30	0.20 ± 0.21	0.02*
13–15 yrs	67 (42.1%)	0.99 ± 0.47		0.29 ± 0.14	
16–19 yrs	44 (27.7%)	0.99 ± 0.42		0.34 ± 0.28	
**Visual impairment**					
Category I	69 (43.4%)	0.99 ± 0.43	0.51	0.27 ± 0.24	0.41
Category II	90 (56.6%)	0.92 ± 0.46		0.28 ± 0.25	
**Overall**	***N* = 159 (100)**	**0.95 ± 0.45**		**0.28 ± 0.25**	

^*^p ≤ 0.05

### Dental caries

The overall prevalence of dental caries was 44.7% (*n* = 71), with a prevalence of [42.1% (*n* = 67)] among participants in permanent dentition and [8.2% (*n* = 13)] among those in deciduous dentition. In the case of participants in permanent dentition, dental caries prevalence was slightly higher at [21.9% (*n* = 35)] among male participants compared to female participants at [20.2% (*n* = 31)]. Among the different age categories,there was no significant difference between age and DMFT.Three participants (1.9%) in the permanent dentition had missing teeth secondary to dental caries while no filled teeth were reported across the sample population. The mean DMFT was 0.44 ± 0.60 with the “D” component being higher (0.42 ± 0.50) than the “M” component (0.02 ± 0.14). There was a statistically significant difference between DMFT and sex (*p* = 0.03). Adolescents in category I visual impairment, but in permanent dentition had a mean DMFT of 0.44 ± 0.67 while those in Category II had a mean DMFT of 0.40 ± 0.61. The difference between the mean DMFT in the two categories of visual impairment was statistically significant (*p* = 0.04) (Table 5).

**Table 5:** Dental caries prevalence and experience in permanent and primary dentition

**Characteristic**	**Permanent Dentition**			**Primary Dentition**		
	**Dental caries prevalence**	**DMFT**	***p*-value**^**b**^	**Dental caries prevalence**	**dmft**	***p*-value**^**b**^
**Sex**						
Male	35 (21.9%)	0.42 ± 0.65	0.03*^c^	7 (4.4%)	0.08 ± 0.28	0.53^c^
Female	31 (20.2%)	0.46 ± 0.62		6 (3.8%)	0.08 ± 0.27	
**Age**						
10–12 yrs	28 (17.6%)	0.42 ± 0.20	0.54^c^	9 (7.4%)	0.19 ± 0.39	0.38^c^
13–15 yrs	22 (13.8%)	0.40 ± 0.00		4 (0.8%)	0.06 + 0.24	
16–19 yrs	17 (10.6%)	0.38 ± 0.15		0 (0.0%)	0.00 + 0.00	
**Visual impairment**						
Category I	27 (42%)	0.40 ± 0.67	0.04*^c^	11 (12.3%)	0.01 ± 0.12	0.33^c^
Category II	40 (44.4%)	0.44 ± 0.61		2 (1.0%)	0.13 + 0.34	
**Overall**	**67 (42.1%)**	**0.44 ± 0.60**^**c**^		**13 (8.2%)**	**0.12 ± 0.32 **^**c**^	

*p ≤ 0.05

^b^Difference in DMFT/dmft values

^c^Bonferroni correction test

In the deciduous dentition, dental caries prevalence was [4.4% (*n* = 7)] among male children and [3.8% (*n* = 6)] among female children. The mean dmft was 0.12 ± 0.32, composed solely of the “d” component.There was no significant difference in dmft scores between sex, age and visual impairment (Table 5). A mild correlation was found between dmft and plaque score index (rs = 0.32, *p* = 0.04).

## Discussion

The goal of this study was to determine the oral health status and oral hygiene practices among visually impaired adolescents attending the largest public primary boarding school for the blind in Kenya. It was hypothesized that good oral hygiene practices were associated with a lower prevalence of oral diseases. This study aimed at determining the prevalence of dental caries and gingivitis, evaluating oral hygiene status, investigating oral hygiene practices, and determining the association between oral hygiene practices and oral health status. In the current study, we found gingivitis to be highly prevalent (88.1%), almost half of the study population to be affected by dental caries (44.7%), and the frequency of toothbrush replacement to be significantly associated with age and gender. Null hypothesis was tested for association between oral hygiene practices and oral health status. Oral hygiene practices did not influence oral hygiene status and dental caries status, however, an association was reported between frequency of toothbrush replacement and gingival index score (“*p*” =< 0.001). The study participants were grouped into Category I (43.4%) and II (56.6%) visual impairment and educated using Braille [20]. In our study, the Central Region of the country had the largest representation (32.7%) of the study population, which may have been attributed to the geographic proximity of the region to the school. Nairobi, the capital city of Kenya, had the lowest representation (12.6%) among the regions bordering the school. This was perhaps due to its higher concentration of schools with integrated learning that include special units for visually impaired children [20]. This is indicative of a school model that could be expanded to other regions for the benefit of the visually impaired and could be a relief to the burden of education for the Central Region.

We found that all the participants in the study brushed their teeth using commercial toothbrushes and toothpaste (99.4%), a finding that is similar to previous studies by Azrina [21] and Ali [22]. Even though the majority of our surveyed children (93.1%) did not know if the toothpaste they used contained fluoride, most of the commercially available toothpastes in Kenya are fluoridated, and hence the deduction that most of the children could be experiencing the protective benefit against caries conferred by fluoride. In the current study, most (67.3%) participants brushed two or more times daily, perhaps tooth brushing habits could be attributed to the institutionalized nature of the school, providing standardized enforcement of oral hygiene measures. This attribute is in line with the WHO guidelines where instilling school oral health programs and preventive habits like daily tooth brushing in children, are advocated [23].

We also found that study participants used adjunct devices in cleaning their teeth. Modified wooden toothpicks obtained from trees within the school compound had the highest application (62.9%) of all the devices. These results contrast with those in a study carried out in Malaysia where the use of conventional toothpicks was low (14.9%) [21]. Almost half (42.7%) of the participants in the current study used “Mswaki”, a traditional toothbrush made from tree twigs. “Mswaki” trees have been reported to have antibacterial properties and may have contributed to the low prevalence of dental decay in the study [24]. In the current study, 41.5% of the participants replaced their toothbrushes at 3 months. Socioeconomic factors as well as the lack of knowledge of ideal oral hygiene practices may have contributed to not changing toothbrushes according to the recommended 3-month timeline. In contrast, a Malaysian study reported 30% of the study participants changed toothbrushes before 3 months, raising a concern that they may have been employing incorrect brushing techniques [21]. In this study, gender was shown to influence the frequency of toothbrush replacement (*p* = 0.04), with more female participants replacing toothbrushes at 3 months. It has been suggested that women practice stricter hygiene norms compared to men, and this might have informed their decision on toothbrush replacement [25]. An association was also reported between toothbrush replacement and age (“*p*” =  < 0.001), with older children more likely to change toothbrushes at 3 months compared to younger children. Possibly, the level of psychological development could have influenced this. Other observations were that most participants (86.1%) rinsed their mouths with water after meals, a practice that could aid in cleansing the oral cavity.

The mean plaque score for the participants was 0.95 ± 0.45 depicting good oral hygiene. Good oral hygiene could have been associated with the frequency of tooth brushing, with the majority (67.3%) of the participants reporting brushing two or more times daily. This finding is comparable to results obtained in other studies [12, 26] but differed from several other studies where fair to poor oral hygiene among visually impaired individuals were observed [11, 27, 28]. Perhaps, the adjustment in behavior where most participants deliberately rinsed their mouth prior to the dental examination could have also affected the oral health outcome reported here.

Despite the children having a high prevalence of gingivitis (88.1%), the mean gingival score was low (0.28 ± 0.25), indicative of mild gingival disease. These findings contrast with other studies where moderate to severe gingivitis was reported [5, 29]. The gingival score index was influenced by the participant’s age (*p* = 0.02), indicating a possibility that severity of gingivitis differed across age categories. This could have been due to the child’s transition into adolescence, a phase where children tend to lack consistency in oral hygiene practice as instructed by their caregivers, and the influence of sex hormones in the pathogenesis of periodontal disease in the peak age (12 years for females and 13 years for males) [30]. Plaque score also significantly (*p* =< 0.001) influenced the gingival score.

In the current study, the overall prevalence of dental caries was (44.7%), (42.1%) among participants in permanent dentition and (8.2%) among those in deciduous dentition.These findings varied with a study in Khartoum State, Sudan where the overall prevalence of dental caries was (46.8%), (19.6%) among participants in permanent dentition and (23.9%) among those in the primary dentition [11]. A slightly higher dental caries prevalence was reported among female participants in permanent dentition compared to their male counterparts (*p* = 0.03). This aligns with a previous study in China [31], where the prevalence of dental caries was higher in girls than in boys (*p* < 0.05). Higher dental caries rate in women has been postulated as multifactorial, caused by social factors, hormonal changes, differing salivary composition and flow rate, and variants of the AMELX gene [32, 33]. No restored teeth were reported in this study suggesting a high unmet treatment need, with participants suffering from dental decay receiving dental extraction as the treatment option. Among participants in permanent dentition,visual impairment was also shown to influence dental caries experience with a higher disease burden reported among participants in Category II (*p* = 0.04). In contrast, other studies did not report a significant association between dental decay and visual impairment [12, 27, 34].

## Conclusions

In general, the participants in this study had fair oral hygiene with gingivitis being highly prevalent (88.1%), and almost half of the study population suffering from dental caries (44.7%). The frequency of toothbrush replacement was significantly associated with age and gender. The majority of the participants were unaware of using fluoridated toothpaste or the need to change toothbrushes within 3 months. Oral hygiene practices did not influence oral hygiene status and dental caries status. However, an association was reported between frequency of toothbrush replacement and gingival index score. The study findings provide evidence for policy change that can go to the incorporation of an expanded school model for the visually impaired to other regions of the country to relieve the burden of education off the Central Region. This would ensure oral health education is instituted in the schools to cater to visually impaired children, cognizant of age and its role in the ideal practice of oral hygiene. The high unmet treatment need should inform the formulation and implementation of preventive oral health school programs and continuous screening assessments with the aim of early diagnosis and treatment of oral disease. Guidelines on maintenance of oral hygiene should also be formulated and continuously communicated to visually impaired children with special consideration to involve both tactile and verbal communication. A comparative case–control study with sighted peers, children with other categories of visual impairment and children from other schools for visually impaired is recommended. A longitudinal study is further recommended to assess oral hygiene practices over time.

## Data Availability

The datasets used and/or analyzed during the current study are available from the corresponding author on reasonable request.
